# Coming full circle: On the origin and evolution of the looping model for enhancer–promoter communication

**DOI:** 10.1016/j.jbc.2022.102117

**Published:** 2022-06-09

**Authors:** Tessa M. Popay, Jesse R. Dixon

**Affiliations:** Gene Expression Laboratory, The Salk Institute for Biological Studies, La Jolla, California, USA

**Keywords:** 3D genome, Cohesin, CTCF, chromatin structure, genome structure, TAD, transcription, transcription enhancer, promoter, AID, auxin-inducible degron, E-P, enhancer–promoter, eRNA, enhancer RNA, LCR, locus control region, LPS, lipopolysaccharide, NPC, neural progenitor cell, TAD, topologically associating domain

## Abstract

In mammalian organisms, enhancers can regulate transcription from great genomic distances. How enhancers affect distal gene expression has been a major question in the field of gene regulation. One model to explain how enhancers communicate with their target promoters, the chromatin looping model, posits that enhancers and promoters come in close spatial proximity to mediate communication. Chromatin looping has been broadly accepted as a means for enhancer–promoter communication, driven by accumulating *in vitro* and *in vivo* evidence. The genome is now known to be folded into a complex 3D arrangement, created and maintained in part by the interplay of the Cohesin complex and the DNA-binding protein CTCF. In the last few years, however, doubt over the relationship between looping and transcriptional activation has emerged, driven by studies finding that only a modest number of genes are perturbed with acute degradation of looping machinery components. In parallel, newer models describing distal enhancer action have also come to prominence. In this article, we explore the emergence and development of the looping model as a means for enhancer–promoter communication and review the contrasting evidence between historical gene-specific and current global data for the role of chromatin looping in transcriptional regulation. We also discuss evidence for alternative models to chromatin looping and their support in the literature. We suggest that, while there is abundant evidence for chromatin looping as a major mechanism for enhancer function, enhancer–promoter communication is likely mediated by more than one mechanism in an enhancer- and context-dependent manner.

Genetic sequences capable of regulating transcriptional output in *cis* from a location distal to a promoter were first reported in the early 1980s and were given the name “enhancer” elements (1, 2, 3). The defining genetic characteristics of enhancers were orientation independent, acting in *cis*, and the ability to influence transcription over large distances (4). Enhancers form part of the intricate machinery that regulates spatiotemporal gene expression patterning and enables cell differentiation and specialization. Enhancers are generally short (100–1000 bp) regions rich in DNA elements that can be bound by transcription factors for the recruitment of chromatin modifying and remodeling complexes, coactivator proteins, and ultimately the general transcription machinery (5, 6). Active enhancers are primarily marked by monomethylation of H3K4 and acetylation of H3K27, the histone acetyltransferase p300, the transcriptional coactivator complex Mediator, RNA polymerase II, and PolII-transcribed enhancer RNAs (eRNAs) (6). Enhancers affect multiple stages of transcription, including both initiation and elongation (7, 8, 9), but depend almost exclusively on their ability to directly communicate with nearby promoters to enact transcriptional control. While there was no obvious initial mechanism for how such elements might affect their distal promoters, it became apparent early in the study of enhancers that structural mechanisms must exist to link enhancers to promoters. For example, Grosschedl and Birnstiel (10) posed that transcription may be affected “at the level of chromatin assemblage and structure,” with the authors emphasizing that the “eukaryotic promoter would then have to be viewed as a 3D, rather than a linear, chromosomal structure”.

More than 2 decades following the identification of enhancer elements in the genome, the physical structures that may facilitate enhancer–promoter (E-P) communication have come into clearer focus. *In vivo*, chromatin is structured into multiple layers of organization, including chromosome territories, active and inactive chromatin compartments, topologically associating domains (TADs) and chromatin loops (11, 12). Close spatial proximity of two genetically distant loci, with exclusion of intervening DNA, is the basis of a chromatin loop. TADs represent segments of the genome that favor, and perhaps restrict, intrachromosomal interactions, including chromatin loops. Recent work has also begun to uncover the mechanisms and protein complexes that give rise to such 3D genome structures, facilitating experiments to more directly test the role of chromatin looping in gene regulation (13, 14). Despite the dominance of the looping model for E-P communication, these recent studies have shown that the contribution of the 3D genome structure to steady-state transcription appears minimal (13, 14). This has raised questions on what relevance, if any, looping has to transcriptional regulation and has prompted the emergence of alternative models through which enhancers could influence promoters (15, 16). However, recent evidence has also indicated that chromatin looping is critical for gene activation, rather than for constitutive expression, suggesting that the role of looping in gene regulation may be context dependent (17). Even with this reassurance, it is evident that looping may not be the sole mechanism for E-P communication. Indeed, the story of looping is bookended by alternative models that have disappeared or emerged during its tenure.

In this review, we attempt to synthesize classical observations of chromatin looping and enhancer function with more recent studies on the mechanisms that govern chromosomal looping and the effects of perturbing the chromatin looping machinery on gene expression. We start by reviewing the historical evidence that contributed to the rise of the looping model and discuss other early alternative models for E-P communication. We also discuss prior gene specific data that established evidence for chromatin looping *in vivo* and its causal role in transcription regulation. Subsequently, we review more recent data on the mechanisms that give rise to chromatin loops and discuss the results of experiments perturbing the looping machinery and their effects on gene regulation that have raised potential questions regarding the role of looping in gene regulation. Throughout this review, we try to synthesize both historical data and more recent observations to obtain a more holistic view of the role of chromatin looping in enhancer function. Indeed, we address here the possibility that 3D architecture is primarily important in mediating changes in transcriptional state and how we may utilize this concept to more appropriately interpret past data and guide the collection of future data.

## On the origin of the looping model

**Figure 1:**
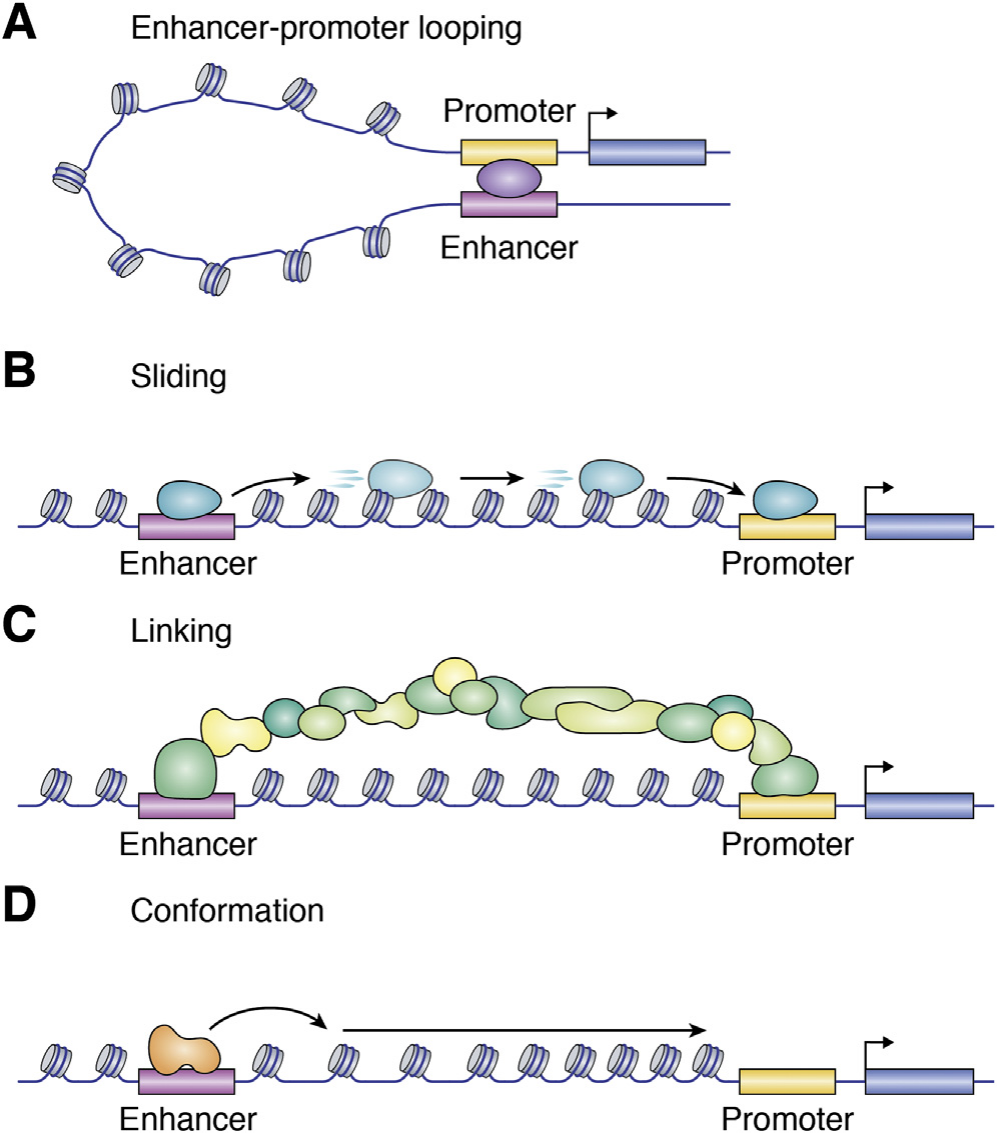
**Mechanisms to enable enhancer-promoter communication.***A*, a tethering protein supports close spatial proximity of the enhancer and promoter, with the intervening chromatin maintained in a loop structure. *B*, in the sliding model, a protein initially associates with the enhancer before translocating along DNA to the promoter. *C*, to link an enhancer with its promoter, protein binding to the enhancer may initiate formation of a protein bridge to transmit signals to the promoter. *D*, changes in chromatin conformation are propagated from the enhancer to the promoter, leading to alterations in the local promoter structure.

DNA looping was initially proposed as a means by which DNA-bound regulatory proteins could access a non-adjacent target sequence. Originally referred to as “direct ligand transfer” (18) or “intersegment transfer by ring closure” (19, 20), these looping models proposed that two regions of DNA come together transiently *via* a protein bridge to enable transfer of protein from one region to the other. With the identification of distal, *cis*-acting regulatory elements in both prokaryotes (21) and eukaryotes (10), the looping model was repurposed, albeit arising apparently independently, as a mechanism by which distal elements could affect transcription (22, 23, 24, 25) (Fig. 1*A*). At the same time, several alternative non-looping models were suggested to explain enhancer function. “Sliding” (also “tracking” or “scanning,” Fig. 1*B*) was proposed as a potential mechanism by which the SV40 enhancer affects transcription by serving as a “bidirectional entry site” for RNA polymerase II to track or scan along chromatin until it reaches an initiation site (4). A structural “linking” model proposed that communication between the enhancer and promoter occurred through formation of a protein scaffold (Fig. 1*C*). In addition, “conformation” models proposed that conformational changes in DNA initiating at the enhancer could transmit a signal to the promoter region, possibly involving propagation of decompacted chromatin (Fig. 1*D*) (26). The SV40 enhancer was also suggested to potentially affect localization within the nucleus, by directing “the DNA template into a specialized nuclear compartment containing all factors required for efficient transcription” (4). The ongoing relevance and reincarnations of all of these models, including those that faced initial skepticism, will be addressed later in this review.

Since its initial proposal, the looping model for E-P communication emerged as a clear favorite (26, 27), driven by accumulating structural and functional evidence. Loop-like structures had been observed in cells, including the late 19th century observation of lampbrush chromosomes in most non-mammalian oocytes (28) as well as in histone-depleted interphase cells by electron microscopy (29, 30, 31), with estimated loop sizes ranging from 30 kb to 100 kb (31, 32). Functional evidence also emerged to suggest a role for looping in regulation by distal sequence elements, originally derived from studies of the *araBAD* operon in *Escherichia coli* (22) and the SV40 enhancer (33). Specifically, perturbing the helical phasing between binding sites for regulatory regions and their non-adjacent promoter could affect transcriptional output (22, 33), which suggested that the orientation of these elements and their binding proteins in 3D space was critical for function. This concept was further emphasized by Hochschild and Ptashne (34), who demonstrated cooperative binding of λ repressor to a pair of operators only if an even number of helical turns was present between binding sites, and subsequently, imaged these helical phase-dependent loops *in vitro* using electron microscopy (35). Perhaps the most compelling evidence, however, emerged from studies showing that enhancers on exogenous plasmids can act in *trans* but only if artificially brought into close proximity through using either a biotin-streptavidin protein bridge (36) or plasmid catenation (37). These early data provided strong support for the hypothesis that chromatin looping, or at least close spatial proximity, between an enhancer and a promoter was sufficient for transcriptional activation. As a result, the evidence in favor of looping appeared definitive: non-histone proteins mediate loop formation (29, 30, 31), certain proteins can induce chromatin looping *in vitro* (35), mutations that perturb *in vitro* looping also perturb transcriptional activation *in vivo* (38, 39, 40), and close E-P spatial proximity is sufficient for transcriptional activation (36, 37). As a consequence, looping became widely regarded as the most likely mechanism by which enhancers influence promoter function from a distance (26, 27, 41).

### *In vivo* evidence of looping

Following the observations of DNA looping *in vitro*, the first structural and functional evidence of E-P looping *in vivo* were derived from studies of a classic model for gene regulation, the β-globin locus. β-like globins are expressed in a developmental stage specific manner, where a transition from fetal β-like globins to adult β-globins occurs shortly after birth (for review of the β-globin locus and the role of looping in its regulation see Sankaran and Orkin (42) and Cavazzana *et al*. (43)). The control of expression of β-like globins is mediated by a locus control region (LCR), located ∼50kb upstream of the embryonic globin gene in humans (44, 45). The LCR contains what was originally described as a cluster of “superhypersensitive” sites (44) that are sufficient to direct the correct level of stage-specific expression of the globin genes (44, 45).

Chromatin looping was proposed as an early model for how the LCR could achieve stage-specific regulation of globin genes (46). However, the development of the novel chromosome conformation capture (3C) (47) and “RNA-trap” (48) methods were required to directly observe looping *in vivo*, showing that the LCR contacted the β-globin genes only in erythroid lineages expressing globin (48, 49) and in a developmental stage–specific manner (50) (for review of current techniques for studying chromatin interactions, see Kempfer and Pombo (51)). Beyond the structural evidence, the endogenous β-globin locus was also used to demonstrate the causal role of looping to transcriptional regulation *in vivo*. Zinc finger-mediated tethering of the LCR to the β-globin promoter is sufficient to promote transcriptional activation of an otherwise inactive gene (52, 53). Similar observations have been made with transient, forced loop formation at additional loci using CRISPR/dCas9–based systems and inducible dimerization systems (54, 55). Taken together, these forced looping experiments demonstrate that, at least at a select set of genomic loci, chromatin looping can play a causal role in gene activation. However, in order to determine whether looping as a model for E-P communication is generalizable to a genome-wide scale would require a much broader understanding of the mechanisms that contribute to chromatin loop formation in the genome, an area of understanding that has rapidly developed over the last decade.

### CTCF and Cohesin as structural determinants of looping

How loop formation occurs was among the more pressing questions arising from the observation of physical contact between regulatory regions and associated genes (56). While E-P communication could occur *via* passive mechanisms such as diffusion, this has often been discounted as a means of loop formation, particularly over long genomic distances. This is in part due to the fact that DNA undergoes subdiffusive motion, meaning that two genomic loci if initially separated by large 3D space are very unlikely to come in contact, in particular at short timescales (57). Riggs (58) surmised such future observations, questioning whether “a complicated folding pattern [can] survive 10^16^ mitoses without a folding-repair mechanism? Random diffusion as a repair mechanism for relatively large structures separated by 100 kb intuitively seems inadequate.” Consistent with this concept, in the decade following identification of *in vivo* looping at the β-globin locus, proteins pivotal to E-P looping and 3D genome organization emerged, centered almost exclusively on Cohesin and its associated proteins (59, 60, 61, 62, 63, 64, 65, 66).

**Figure 2:**
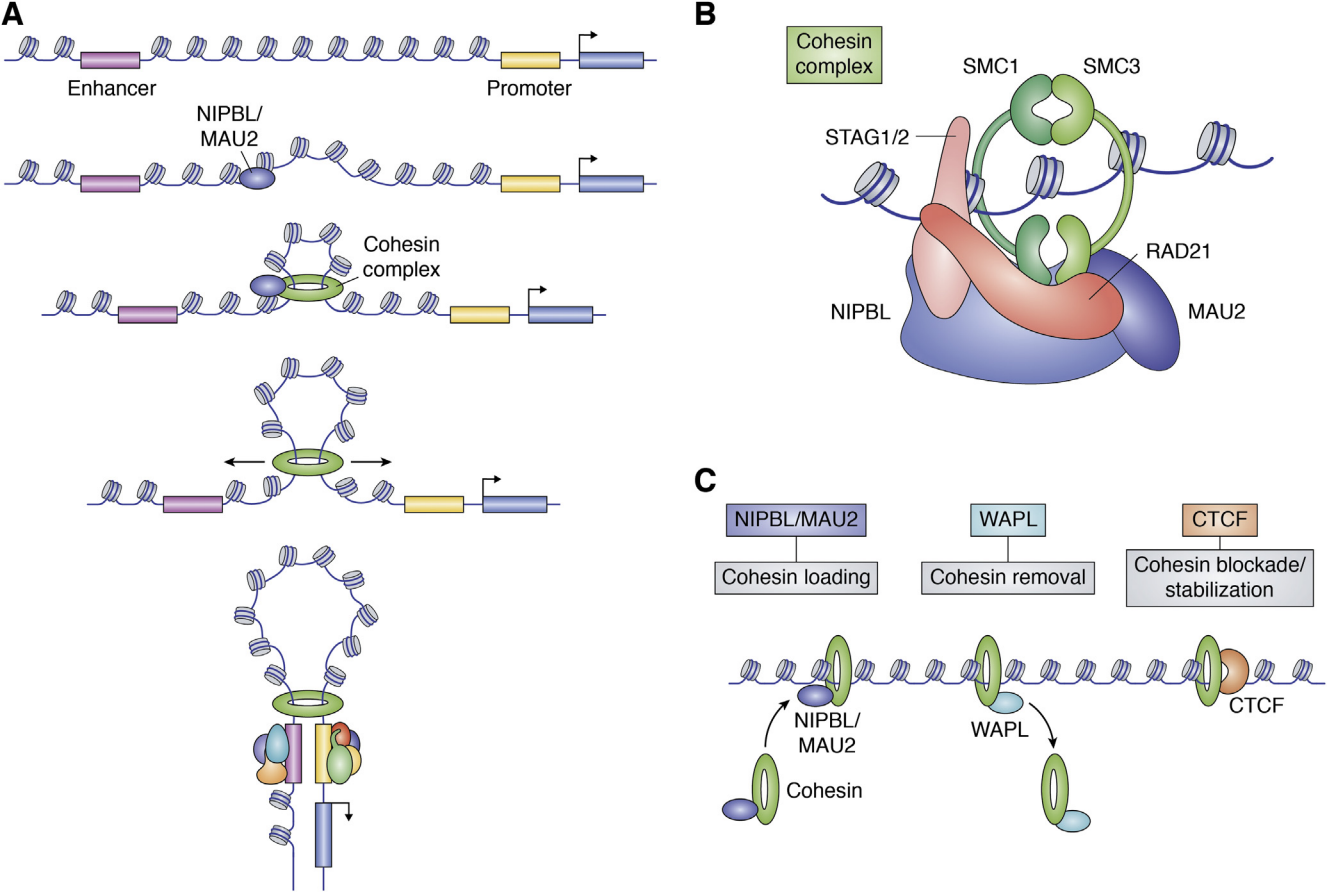
**TheCohesin complex as the mediator of the loop extrusion process.***A*, the ring-like Cohesin complex (*green*) is loaded onto chromatin through the activity of NIPBL/MAU2, with Cohesin subsequently translocating outward, bringing the enhancer and promoter into close spatial proximity. *B*, components of the Cohesin complex. The core complex members are RAD21, SMC3, SMC1, and one of the mutually exclusive STAG proteins. NIPBL and MAU2 are primarily thought to contribute to loading Cohesin onto DNA. *C*, Cohesin depends on a number of proteins to enable its function on chromatin. The primary known factors responsible for Cohesin function are NIPBL/MAU2 for Cohesin loading, WAPL for Cohesin removal, and CTCF for blocking Cohesin translocation and stabilizing it on chromatin.

Cohesin is a protein complex consisting of SMC1, SMC3, RAD21, and either STAG1 or STAG2 (Fig. 2*B*) and was initially identified for its role in sister chromatid cohesion (67, 68). The function of Cohesin is supported by at least Nipped-B-like (NIPBL) and MAU2 as loading factors, WAPL as a release factor, and the DNA-binding protein CTCF as an insulator (Fig. 2*C*) (69). The core Cohesin complex members SMC1 and SMC3 were the first to be attributed a role in 3D genome organization (59). The global influence of Cohesin and CTCF on genome organization was made evident as the result of Hi-C studies (70), allowing for genome-wide characterization of structural features of genome organization, including chromatin compartments (70), TADs (11, 12, 71), and chromatin loops (72). These studies showed that most TAD boundaries and chromatin loops are occupied by CTCF and Cohesin, suggesting that these were critical factors in loop and TAD formation. Furthermore, Cohesin dysfunction has been linked to human diseases through syndromes resulting from mutations in complex components, termed Cohesinopathies (for review, see Piché *et al*. (73)), suggesting that Cohesin-mediated looping may play critical roles in regulating gene expression in development.

From the proposed role of Cohesin and CTCF in TAD and loop structure, genome-wide assays could be used to evaluate the consequences of their perturbation. The application of the auxin-inducible degron (AID) system for acute degradation led to the finding that loss of Cohesin causes a near-complete disruption of chromatin loops and domains (14, 74). Similar observations have also been made in *Rad21* conditional KO systems (75). Application of the AID system to CTCF caused a similar loss of loops and resulted in reduced TAD boundary integrity (13, 74, 76, 77, 78). These results demonstrated conclusively that CTCF and Cohesin are responsible for the vast majority of chromatin loops observed in the genome.

### Mechanism of loop formation

While it was evident that CTCF and Cohesin were responsible for most loops and TADs in the genome, it was unclear how these proteins gave rise to such structural features. Mechanistic insights into CTCF and Cohesin function in TAD formation were spurred by the observation that the majority of chromatin loops that demarcate TADs contain convergent CTCF-binding sites at their anchors (11, 72). Further, inversion of CTCF-binding sites caused a significant reduction in loop formation (79, 80, 81). These observations led to an intense search for mechanistic models that could account for such motif orientation rules. What emerged was the now extensively studied “loop extrusion” model, which bore similarities to early “processive” or “tracking” models for the formation of chromatin loops, such as the “DNA reeling” model proposed by Riggs (58), “associative tracking” by Woods and Tonegawa (82), and “facilitated tracking” by Blackwood and Kadonaga (83) (Fig. 2*A*). With regards to the formation of chromatin loops, the loop extrusion model posits that Cohesin will “extrude” a loop of chromatin until it is blocked by an appropriately oriented CTCF-binding site (Fig. 2*C*). The loop extrusion model, albeit without regard to enhancer function, was proposed by Nasmyth (84), and again by Alipour and Marko (85), as a mode of action for the ring-like, condensin complex. While condensin has since been implicated in loop extrusion in the context of chromosome condensation for mitosis (86), Cohesin appears to drive a similar process during interphase and was proposed to mediate both TAD formation and chromatin looping through loop extrusion (81, 87).

The specific mechanism of loop extrusion is still unclear but is being intensely investigated. Within Cohesin, SMC1 and SMC3 form the bulk of the ring-like structure and tightly associate at one end to create a hinge-like region. SMC1 and SMC3 have similar structures, with both containing a hinge domain connected to a globular head domain by antiparallel coiled-coils (88). The head domains are capable of binding one ATP molecule each. In the ATP-bound state, SMC1 and SMC3 dimerize at both their hinge and head domains, but in the unbound state, they directly dimerize only at the hinge domain (88, 89, 90). Closure of the ring at the head domain is supported by RAD21, which is associated with multiple Cohesin regulatory proteins, including the core subunits STAG1/2. The Cohesin complex is generally thought to entrap DNA in its ring-like structure (88), but loop extrusion can be driven by entrapment-incompetent Cohesin *in vitro* (91). The extrusion process involves either a single Cohesin complex encompassing two linked stretches of DNA or a dimer of Cohesin complexes each associated with their own DNA molecule and can proceed either symmetrically or asymmetrically from the initial loading site (91, 92, 93, 94). This loading is achieved through the activity of NIPBL (95, 96), whose *Drosophila* homolog, Nipped-B, was first identified for its role in E-P communication and was initially proposed to function in loop stabilization (60). While primarily seen as a loading factor, it has been reported that NIPBL and its cofactor MAU2 are necessary for both Cohesin loading and the maintenance of loop extrusion (91). The extent of Cohesin-dependent loop extrusion is dictated by CTCF, a transcription factor that is associated with barrier and insulator elements in the genome (97). While not absolutely clear how CTCF constrains Cohesin movement along chromatin, one recurring proposal is that it functions as a physical blockade to Cohesin translocation, with a recent paper suggesting that the N terminus of CTCF is necessary for Cohesin stalling (98). In addition to CTCF, the extent of loop formation is controlled by the Cohesin-removal factor WAPL (99). Indeed, the actions of WAPL appear to be blocked by correctly oriented CTCF, the N terminus of which is able to associate with Cohesin through the same region as WAPL, thereby creating a competitive-binding module (100).

The capacity of Cohesin to translocate along chromatin means that it is a system that is built to move. However, it is evident from Hi-C that the 3D architecture does maintain a level of stability, as demonstrated by recurrent structural features that are visible in maps. While we know CTCF contributes to at least part of this stability, it is insufficient to explain the entirety of the observable 3D architecture. eRNAs, for example, have been found to stimulate E-P looping (101, 102, 103) and in a number of cases are additionally reported to associate with Cohesin and promote its recruitment to enhancers (104, 105). In general, the effect of eRNAs on these processes occurs in *cis*, but in at least one instance, eRNAs influence Cohesin in *trans* at unlinked promoters (106). Through this mechanism, eRNAs can provide a level of specificity for Cohesin-mediated loop formation, although largely from the perspective of the enhancer itself. On the other hand, CTCF has also been found to influence E-P loop selection by binding with variable strength to the promoter, a role that is distinct from controlling TAD formation (107). Indeed, through this mechanism, CTCF can guide the selection of a promoter to form a loop with a nearby, TAD-restricted enhancer (107). Another identified regulator of E-P looping is the transcription factor ZNF143, which was found to be enriched at loop anchors and strongly colocalized with CTCF, primarily at enhancers and promoters (108, 109). Indeed, perturbation of ZNF143 specifically disrupts E-P looping and reduces CTCF binding at loop anchor sites (109), suggesting that ZNF143 is a modulator of CTCF recruitment and, as a consequence, likely influences Cohesin-mediated loop extrusion. While our knowledge of determinants of E-P loop formation is limited, one can hypothesize that, due to the dynamic nature of loop extrusion, stable E-P association is perhaps not a necessary prerequisite for functional E-P contact, but a simple “hit-and-run” may be sufficient to induce an appropriate line of communication. Even so, one of the biggest questions of the field is how E-P looping affects transcription.

### Challenging the relationship between the 3D genome and gene regulation

With a greater understanding of the mechanisms and protein complexes that contribute to TAD and loop formation, as well as molecular tools to perturb these protein complexes, the looping model for enhancer function has now been tested by multiple groups in different contexts. These studies have consistently demonstrated that perturbation of Cohesin or CTCF globally has surprisingly modest effects on gene expression. Depletion of the Cohesin subunit Rad21 leads to a total loss of loops and TADs, but the consequences on transcription are minimal, with almost 90% of expressed genes unchanged, and fewer than 75 genes changed more than twofold (14, 78). Disruption of NIPBL, which causes comparable alterations to genome organization as with RAD21 degradation, has also shown a limited number of dysregulated genes (110, 111). Likewise, acute degradation of CTCF has only minimal effects on transcription (13, 76, 78, 112). Some reports have suggested that depletion of WAPL perturbs expression of about 1000 genes (99, 113), but acute WAPL degradation has found that the effects are much smaller on short timescales (78). On the surface, these findings appeared to refute the long-standing hypothesis that enhancer-mediated transcriptional regulation is controlled, at least in part, by E-P looping and, more generally-speaking, that the function of 3D genome structure relates to gene expression. However, these outcomes are also one perspective of an increasingly complex story, whereby context is critical.

Locus-specific genetic perturbations, including structural variants or experimentally induced deletions, have been illustrative of the relationship between 3D genome structure and gene expression, in ways that suggest the context of a given genetic perturbation is critical. Structural variants within the developmentally regulated WNT6/IHH/EPHA4/PAX3 locus that disrupt TAD boundaries lead to perturbation of the 3D genome, ectopic E-P connections, subsequent changes in gene expression, and different phenotypic consequences (114), ostensibly supporting a role for E-P looping in gene regulation. Similarly, structural variants that invert a TAD boundary at the Sox9 and Kcnj2 locus were sufficient to decrease Sox9 expression and increase Kcnj2 expression, likely by altered E-P communication, resulting in limb deformities (115). However, CTCF site deletion at the Sox9–Kcnj2 locus leading to fusion of the Sox9 and Kcnj2 TADs, has only a modest effect on gene expression (115). Consistent with these outcomes, CTCF-binding site inversion has been found to have variable effects on gene expression, including no effect (*Malt1*, *Sox2*), increased (*Fbn2*), and decreased (*Pcdhα*) (79, 80). The effect of naturally occurring structural variants has also been studied in the setting of highly rearranged chromosomes in the context of cancer genomes or *Drosophila* balancer chromosomes, which have shown a minority of structural variants are associated with changes in gene expression (116, 117). For example, structural variants in *Drosophila* balancer chromosomes caused the loss of 12% of TAD boundaries, but only 10% to 12% of genes within close proximity of these boundaries are differentially expressed (116). These results suggest highly context-dependent results of genetic perturbations on 3D genome structure and gene expression, the basis for which remains unclear.

In addition, imaging-based studies have also found mixed results regarding the role of E-P proximity in distal gene regulation. Expression of the developmentally regulated gene sonic hedgehog (*Shh*) is controlled by different enhancers under different contexts, primarily the limb bud-specific enhancer ZRS and the series of brain-specific enhancers SBE. On a correlation basis, *Shh* and ZRS have been found to have close spatial proximity during periods of *Shh* expression (118). In contrast, *Shh* activation during differentiation of mouse embryonic stem cells into neural progenitor cells (NPCs) is accompanied by a reduction in the spatial proximity between *Shh* and the contextually speaking most active enhancer SBE6 (119). Strikingly, synthetic activation of SBE6 using transcription activator-like effector (TALE)–mediated recruitment of VP128 or Mediator was also sufficient to increase the distance between it and *Shh* (119), suggesting that looping, or close spatial proximity, is not part of the mechanisms of action of SBE6 on *Shh* activation.

A similarly unexpected finding was observed by live-cell imaging of *Sox2* and its control region (SCR), which found that close spatial proximity between enhancer and promoter is not a necessary requisite for a transcriptional burst (120). Sequential imaging of nascent RNA and DNA at the bithorax complex (BX-C) of genes in *Drosophila* found a weak correlation between transcription and E-P proximity, including various examples showing the opposite phenotype (*e.g.*, enhancer in close proximity to inactive gene) than what a stable looping model would predict (121). Additional doubt has been cast on the looping model, at least in the sense of a single enhancer associating with a single promoter, with the finding that a single enhancer can simultaneously control the burst profile of at least two reporter genes (122). Thus, while historical evidence favored a role of E-P looping in transcriptional control, there is also an accumulation of contexts in which chromatin looping does not immediately appear relevant to E-P communication. It remains ill-defined when and where looping is relevant for distal gene regulation.

### Loop extrusion for transitions in gene expression

A recurring feature of much of the work suggesting a limited relationship between Cohesin and gene regulation is the focus on steady state transcription. Various studies have, however, observed strong transcriptional perturbation occurs when Cohesin is disrupted during transitions in transcriptional output (*e.g.*, in response to stimuli or gene activation during differentiation). Indeed, this idea aligns well with much of the early evidence supporting a causal relationship between E-P looping and transcriptional regulation, such as the forced looping experiments for transcriptional activation (52, 53). For example, in the context of lipopolysaccharide (LPS)-induced gene expression in quiescent macrophages, deletion of RAD21 disrupted the baseline expression of more than half of the LPS-inducible genes, increasing to 75% of these genes following 8 h of stimulation (17). In the context of neurons, deletion of RAD21 from immature, postmitotic mouse neurons primarily caused dysregulation of neuron-specific genes, including a preferential effect on activity-dependent genes, over those that are stably expressed (123). Gene-specific studies at the *Shh* locus have shown that depletion of RAD21 resulted in perturbation of distal enhancer-mediated *Shh* activation but not proximal enhancer- or promoter-mediated activation (124). Similar results from Rinzema *et al*. showed that Cohesin was more important to transcriptional activation of a reporter gene when the E-P distance was greater, in contrast to Mediator and GATA, which contribute to transcriptional activation independent of the E-P distance (125). Antony *et al*. have also found that depletion of STAG2 impairs enhancer-dependent control of gene expression, specifically of RUNX1 and ERG, following induction of megakaryocyte differentiation of K562 cells (126). Taken together, these results have shown that Cohesin is required for many transitions in gene expression in response to stimuli or differentiation cues.

One possible mechanism by which Cohesin might preferentially contribute to transitions in gene expression is through its rapid turnover from chromatin, which would enable changes in E-P communication. Consistent with this, depletion of the Cohesin removal factor WAPL or the core subunit RAD21 show surprisingly similar patterns of upregulated and downregulated genes (127), suggesting that it is a Cohesin loading/unloading cycle that is the critical component of looping-mediated transcriptional control. In addition, the subunit composition of Cohesin may influence its likelihood for stable *versus* dynamic looping. Wutz *et al*. (128) found that the majority of Cohesin^STAG1^ and Cohesin^STAG2^ complexes have similar residence times (∼7–15 min), but a subset of Cohesin^STAG1^ remains on chromatin for around 5 h. Based on their contrasting residence times, one would expect the long-lived Cohesin^STAG1^ subset to modulate stable loops, whereas the more transient Cohesin^STAG1^ and Cohesin^STAG2^ would both contribute to dynamic loops. In general, STAG2-containing Cohesin is preferentially distributed at enhancers and promoters that lack CTCF-binding sites, whereas STAG1-containing Cohesin tends to be associated with TAD boundaries, which are enriched for CTCF-binding sites (129). Dynamic Cohesin could then be a relevant medium through which enhancer-dependent transcriptional control could be rapidly transmitted to promoters.

The role of CTCF in transitions in gene expression is more context dependent. For example, CTCF depletion in LPS-stimulated macrophages also appears to affect the expression of the majority of induced genes (130, 131). Additionally, Kubo *et al*. (132) determined that the depletion of CTCF perturbs the differentiation of embryonic stem cells to NPCs, with genes linked to neural differentiation most affected by CTCF depletion. In contrast, transdifferentiation of B-cells into macrophages is apparently unaffected by CTCF depletion, despite this causing widespread perturbation of 3D structures (131). Distinguishing the circumstances where CTCF impacts transitions in gene expression from those where it does not, will be an important issue to address in the future.

**Figure 3:**
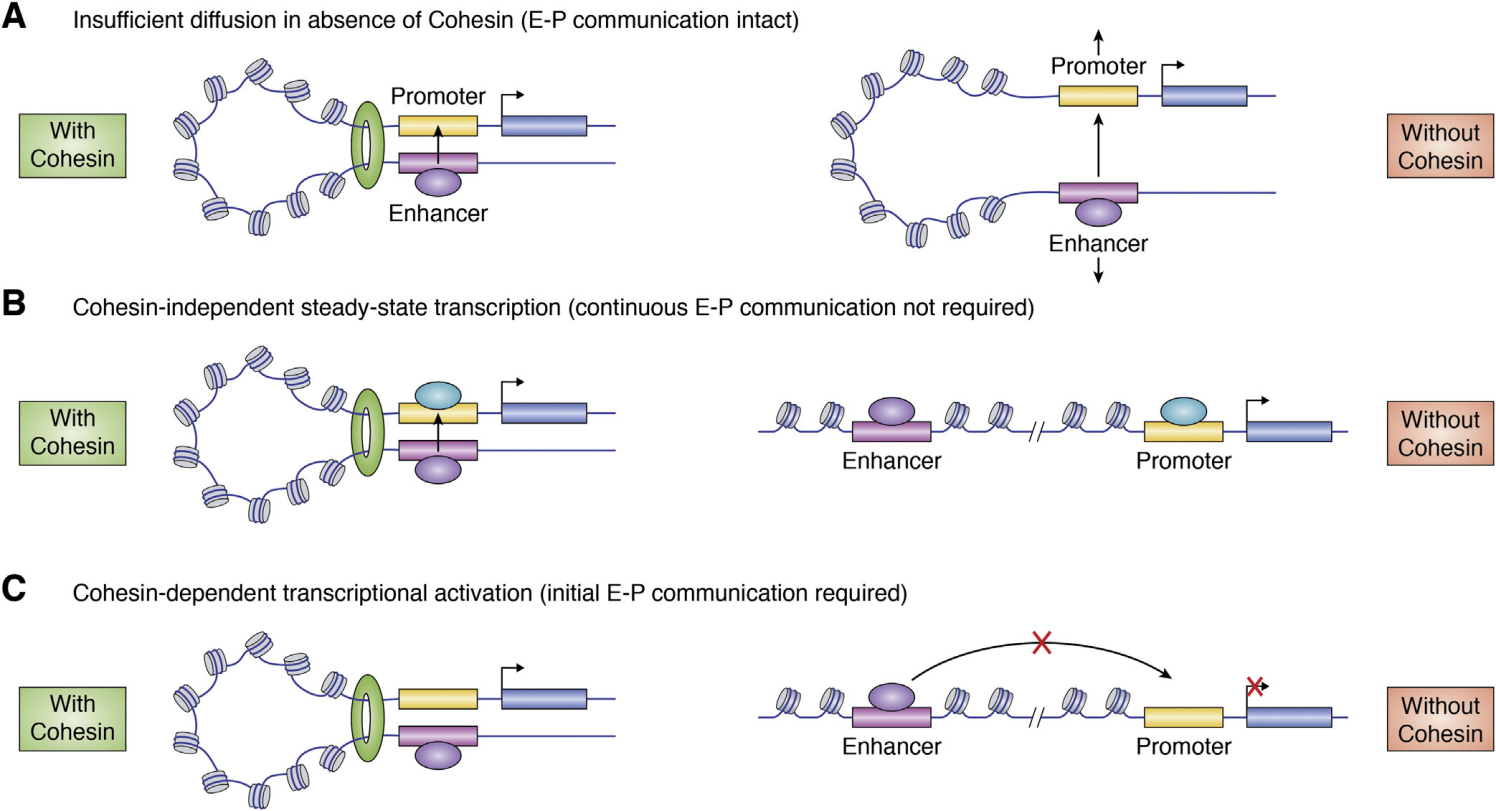
**Scenarios enablingCohesin-dependent andCohesin-independent transcriptional regulation.***A*, E-P loops remain intact following removal of Cohesin, due to either limited diffusion or an additional tethering factor maintaining the loop structure. *B*, transcriptional activation requires only initial E-P looping, such as that required to transfer transcriptional machinery from the enhancer to promoter. When Cohesin is absent, the loop structure is lost, but the constitutive transcription is retained. *C*, for a subset of genes, Cohesin and loop formation are necessary prerequisites for transcriptional activation. This could mean that Cohesin promotes loop formation in response to an activating signal or that loop formation enables the activating signal to be transmitted. E-P, enhancer–promoter.

Why does perturbation of 3D architecture have such a limited effect on transcription at steady state but a more pronounced effect on gene expression transitions? One possibility that has been raised in response to the minimal effect of Cohesin/CTCF depletion on transcriptional output is that while the dominant structures are eliminated by CTCF or RAD21 degradation, less prominent interactions between enhancers and promoters may retain connectivity, at least at short timescales. Specifically, RAD21 depletion appears to have a more limited impact on E-P communication, especially at early time points, and that a large number, albeit the minority, of promoter interactions are maintained in the absence of Rad21 (78, 133). This maintained E-P connectivity may be attributable to either minimal free diffusion following loop formation or additional stabilizing factors (Fig. 3*A*). This may suggest that loop formation initiates a self-perpetuating transcriptional cycle whereby relaxing of the loop, through either innate processes or artificial disruption of looping machinery, has only minimal consequences on recruitment of the transcriptional machinery to the promoter and ongoing gene expression (Fig. 3*B*). This process is unlikely to be feasible for inducible genes and implies looping remains necessary for initiating transcription of at least a subset of constitutive genes. In the former context, a stimulus may promote loop formation (*e.g.*, by favoring NIPBL-mediated Cohesin loading) or enable transcriptional activation following loop formation (Fig. 3*C*), with both of these requiring functional looping machinery. The dynamicity of the majority of Cohesin complexes therefore could create an environment in which transcriptional change is easily accessible.

### Non-Cohesin looping factors

Much attention has been paid in recent years to the role of Cohesin and CTCF in the formation of chromatin loops in the genome. In light of the minimal effects of CTCF or Cohesin depletion on steady-state transcription, it is important to consider that several non-CTCF/Cohesin proteins have also been implicated as contributing to loop formation either independently or coordinately with Cohesin and CTCF. As the list of non-CTCF/Cohesin looping factors could be quite long with varying levels of support in the literature, we will specifically discuss here factors that we believe have the strongest evidence for a role in E-P looping in mammalian organisms.

One of the better characterized non-CTCF/Cohesin looping factors in mammals is the LIM domain–binding family protein Ldb1. Though lacking direct DNA-binding activity, Ldb1 binds to LIM-domain transcription factors, such as Lhx2 and Isl-1, as well as other transcription factors and chromatin-associated complexes (134). The *Drosophila* homolog of Ldb1, Chip, was identified in screens of proteins that are required for enhancer function, offering early suggestions that Ldb1 could be required for specific instances of E-P looping (135). Ldb1 self-associates into dimers and higher-order oligomers through a specific dimerization domain that allows Ldb1 to serve as a “molecular bridge” between transcription factors (136, 137). This has been remarkably demonstrated in several systems. Ldb1, in conjunction with Lhx2, is required to form large interchromosomal contacts known as the “Greek Islands” during olfactory neuron development (138). Similarly, through its interaction with Isl-1, Ldb1 is required for proper differentiation of cardiac precursors and regulates chromatin looping at several key cardiac developmental genes (139). Forced tethering of the dimerization domain of Ldb1 at the β-globin promoter is also sufficient to induce chromatin looping to the LCR through dimerization with endogenous Ldb1 (52). These studies all point to key roles for Ldb1 in regulating E-P communication at specific genes in specific cell types. However, it remains unclear if Ldb1 can function as a general looping factor or if it is more specialized for certain genes and cell types.

Another well-studied factor that plays a role in chromatin looping is Ying-yang 1 (YY1). Like CTCF, YY1 is a DNA-binding zinc-finger protein that was originally reported as having either activator or repressor functions depending on promoter context (140, 141, 142). YY1’s role in mediating chromatin looping has been well studied in B-cells (143), where it is essential for B-cell development (144). During B-cell development, YY1 contributes to higher-order chromatin organization of the IgH locus, and YY1 loss has been shown to result in loss of contraction (144) and loss of chromatin looping at the IgH locus (145, 146, 147). Interestingly, more recent work indicates that YY1 may have a more general role in chromatin looping outside of B-cell development. Specifically, Weintraub *et al*. (148) showed by HiChIP that YY1, unlike CTCF, preferentially links enhancers and promoters and that YY1 depletion reduces E-P chromatin interactions genome wide. Similarly, Beagan *et al*. (149) identified a subset of CTCF-independent chromatin interactions in mouse NPCs that were bound by YY1, with YY1 knockdown causing a loss of these long-range contacts. How YY1 contributes to long-range interaction is currently unclear. *In vitro*, YY1 has been shown to have the ability to dimerize or oligomerize (148, 150), while mass spectrometry studies have shown that YY1 can interact with Cohesin and condensin subunits (151). This raises the possibility that YY1 may “co-opt” loop extrusion mechanisms to help facilitate E-P communication.

A final non-CTCF looping factor that will be discussed here is the Mediator complex. In mammals, Mediator is a large 26-subunit complex that directly interfaces with RNA PolII and the general transcription machinery, as has been beautifully illustrated in recent cryo-EM structures (152, 153). Mediator has also been shown to form extensive protein–protein interactions with diverse, sequence-specific transcription factors (154). As a result of the ability to bind to both the general transcriptional machinery as well as sequence-specific transcription activators, Mediator would appear to be a prime candidate for bridging E-P communication (155). Indeed, Mediator binds to many enhancers in the genome (156, 157), and knockdown of Mediator subunits has been observed in several studies to impact E-P interactions at specific loci (156, 157, 158). However, more recent work casts doubt on whether Mediator truly plays an architectural role in the genome (159). Specifically, El Khattabi *et al*. found that degradation of the MED14 subunit led to few clear changes in 3D chromatin architecture. Interestingly, the authors also generated a cell line with a “Tailless” version of Mediator that lacked the MED15, MED16, MED23, MED24, and MED25 subunits. Such cells were viable and showed locus-specific losses of Mediator associated with local changes in 3D genome structure. The authors propose that the distinction is that, over time, the Tailless mutants lead to additional changes in local chromatin states, including local loss of H3K27ac, Cohesin, and CTCF, that is not observed during rapid depletion of Mediator. The authors propose that such changes are a result of long-term dysfunction at these local *cis* elements, and therefore, while long-term depletion of Mediator can lead to changes in 3D genome structure, these effects are indirect. A greater understanding of the functional *versus* architectural roles of Mediator will be important to address in greater depth in the future.

### Alternatives to the looping model for enhancer action at a distance

We believe that there is ample evidence that chromatin looping plays a causal role in gene regulation in certain contexts. However, the modest transcriptional changes observed in recent experiments perturbing Cohesin- and CTCF-based looping (13, 14, 132) and various examples that are inconsistent with looping (119, 120, 121, 131) should lead to a reconsideration of alternative models to looping as a mechanism for enhancer action at a distance. In this section, we will discuss several alternative models that have some level of evidentiary support in the literature. These alternative models do not necessarily have to act in a mutually exclusive manner with looping but instead could act additively or synergistically with looping-based mechanisms.

**Figure 4:**
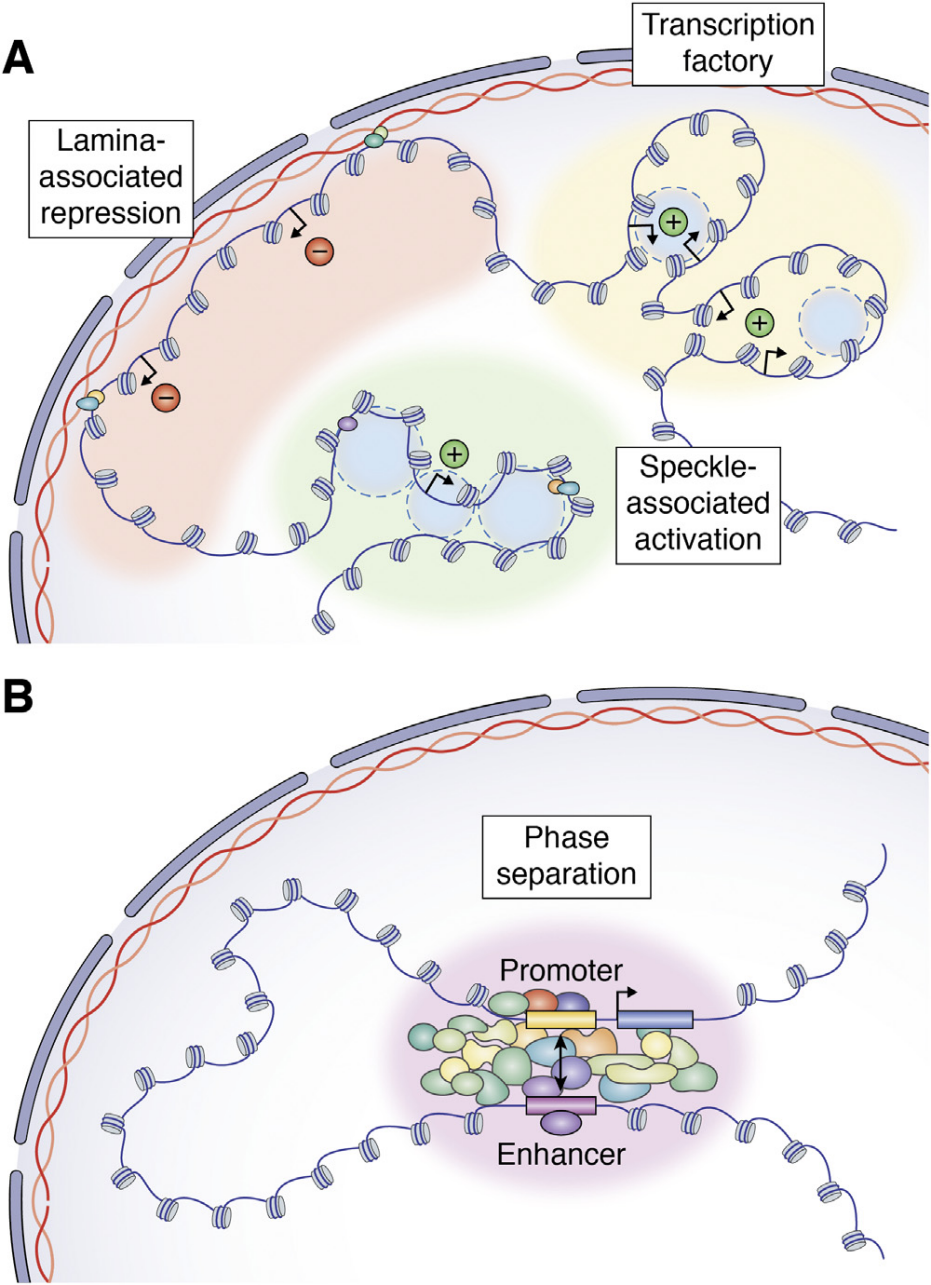
**Non-looping models of enhancer function.***A*, enhancers could play a tethering role to control the distribution of chromatin in the nucleus. For example, regions of chromatin that are tethered to the nuclear lamina by an enhancer may experience transcriptional repression, whereas regions tethered to nuclear speckles are more likely to be activated. Similarly, enhancers may drive the clustering of chromatin into transcription factories, which are environments that are rich in transcriptional regulators and favor transcriptional activation. *B*, phase separation is largely thought to be driven by the formation of large protein clusters. Due to the number of protein-binding sites present in enhancers and promoters, these regions tend to accumulate protein and this may lead to condensate formation, essentially bringing enhancers and promoters into close spatial proximity.

The first alternative model for non-looping enhancer function would be variable localization within the nucleus through association with nuclear bodies or other landmarks. Imaging-based studies have clearly shown that certain loci, when activated or repressed, change position in the nucleus. For example, the HoxB locus moves from a position in the interior to the periphery of its chromosome territory when it becomes activated during embryonic stem cell differentiation (160). Similarly, active genes have been shown to coassociate in 3D space in regions that have been termed “transcription factories” (161, 162). Certain stimulus responsive genes will colocalize upon induced expression, such as by androgen or TNF-alpha signaling (163, 164). Further, forced recruitment to specific nuclear landmarks can affect gene expression. For example, tethering of loci to the nuclear lamina can result in gene repression (165, 166, 167). In this regard, one alternative model for enhancer function would be that some enhancers could directly or indirectly control localization within the nucleus, such that when the enhancer is activated or repressed, it could result in variable association with active or repressive chromatin environments (Fig. 4*A*). Interestingly, when specific loci are tethered to the nuclear lamina, not all nearby genes are repressed (166, 168). This could confer specificity to nuclear relocalization as a mechanism of enhancer function, as not all genes at a locus would necessarily be altered if their localization changed. Recent technology developments may allow for more in-depth characterization of the effects of nuclear relocalization in gene expression. In particular, systems such as CRISPR-genome organization (CRISPR-GO), which enables guide RNA-based targeting of a locus to a nuclear landmark of interest, will facilitate these types of studies (169). Thus far, however, there is not extensive evidence that enhancers or other *cis* elements specifically control nuclear localization, at least independently from other mechanisms for gene activation.

Another alternative to looping as a model for enhancer function is that enhancers control the compaction and decompaction of domains in the genome, therefore allowing for variable access of the transcriptional machinery to neighboring genes. Indeed, this is not dissimilar to the early model of enhancers propagating a signal to nearby promoters (Fig. 1*D*). Repressive chromatin environments, such as H3K27me3 Polycomb sites or H3K9me3/HP1 heterochromatin regions, are in a relatively compacted state (170, 171, 172, 173). It has been postulated that such compaction can directly lead to gene repression potentially by blocking access to regulatory and transcriptional machinery (174). On the contrary, decompaction of specific loci can be observed prior to transcriptional induction (160). Further, compaction can “spread” across domains of chromatin (175). In this sense, if enhancers regulated the accumulation of such repressive chromatin environments, they could modulate the accessibility of distal genes without actually needing to loop in contact with them. This was recently suggested as a mechanism for the activation of the *Shh* gene by a subset of its distal enhancers. Specifically, while the enhancers that regulate Shh expression in the limb, such as the famous ZRS enhancer, show increased association with the *Shh* gene when it is expressed (118), this is not the case for enhancers that regulate *Shh* in NPCs (119). Instead, these elements actually seem to associate less with the *Shh* gene when it is activated in NPCs (119). Such decompaction represents an interesting alternative to looping as a mechanism for distal enhancer activation. However, it is currently unclear to what extent this functions as a mechanism for enhancer action genome wide.

An emerging model for enhancer function that does not require looping *per se* would be the association of loci into phase-separated bodies. There has been tremendous interest in recent years in the role of phase separation in creating membraneless organelles in the nucleus, such as nucleoli or heterochromatin (176, 177). Phase separation has also been proposed as a mechanism for the activation of enhancer elements, in particular strong enhancer elements such as “super enhancers” (16). The general idea is that weak, multivalent interactions between disordered activation domains in proteins and RNAs in the nucleus could lead to bodies that “phase separate” from their surroundings with high local concentrations of activating factors (178, 179) (Fig. 4*B*). If such a phase separated body encompassed both an enhancer and a promoter, then this could lead to gene activation without the gene and enhancer actually having to directly physically contact each other in a loop. Genome-wide evidence for multiway chromatin interactions at potentially phase-separated bodies such as nuclear speckles has recently been observed using the split-pool recognition of interactions by tag extension (SPRITE) assay (180). If phase separation plays a critical role in enhancer function in general, this could possibly explain the modest effects of Cohesin withdrawal on gene expression, as phase separation may still allow for activation of target genes by distal enhancers.

Other potential non-looping models for enhancer function have also been proposed over the years, particularly in the early history of the study of enhancers. The aforementioned discussion is not meant to be an exhaustive list of non-looping models for enhancer function but is meant to highlight specific models that have clear or growing evidence in the literature. We would suggest that such nonlooping-based models warrant further investigation, particularly how such events may act in combination, or in contrast, with more traditional looping-based models.

## Conclusions

Chromatin looping was among the earliest models to account for the observed influence of enhancers over their associated promoters and has persisted as the predominant model through decades of accumulated evidence. This evidence extends from observations of 3D genome structures *in vivo*, including chromatin loops, identification of the major looping machinery, and to demonstrable transcriptional regulation induced by loop formation. The relationship between chromatin looping and the control of gene expression is, however, becoming increasingly complex. Indeed, in studies assessing the functional contribution of chromatin looping to transcriptional regulation, there are distinct classes of evidence; initial *in vivo* challenges found that looping was often sufficient for transcriptional activation, while later global challenges suggested that extensive perturbations in looping had minimal effect on steady-state transcription. We posit that these classes are not incompatible and instead reflect differential contributions to transcriptional initiation and maintenance, with chromatin looping particularly relevant for mediating E-P communication to enable changes in transcriptional state. The terms of this relationship are likely context dependent, but we currently lack the appropriate global studies to understand the specifics of looping-mediated transcriptional activation. Regardless, the path forward must take into account that multiple mechanisms likely bridge enhancers and promoters to enable their communication, with chromatin looping one of many.

### Glossary

**AID** is a system for rapid degradation of a target protein. It involves fusion of a protein of interest to a miniAID and introduction of the E3 ligase complex member OsTIR1 to the cell line (181). Addition of auxin to these cells recruits the miniAID-tagged protein to the E3 ligase complex, initiating its degradation.

**β-globin locus** is region of the genome that encodes the embryonic (ε), fetal (ɣ), and adult (δ and β) β-globin genes. These genes are encoded and expressed sequentially, with the upstream **LCR** regulating their expression *via* loop formation.

**Chromatin conformation capture (3C)** is a family of techniques based on crosslinking, restriction digest, and proximity ligation to identify regions of DNA that are spatially proximal (47). The original 3C technique was based on quantitative PCR using primers for two loci of interest (47) and led to **circular chromosome conformation capture (4C)** for the unbiased identification of interacting loci with a single locus of interest (182), **chromosome conformation capture carbon copy (5C)** for unbiased identification of interacting loci with multiple loci (183), and **Hi-C** for global identification of interacting loci (70). **Capture Hi-C** is a more targeted variant of Hi-C and uses RNA baits to enrich specific loci of interest (184). Another derivative is **HiChIP**, which involves an immunoprecipitation step to identify 3D structures associated with a specific protein of interest (185).

**Cohesin** is a protein complex that includes **SMC1**, **SMC3**, **RAD21**, and either **STAG1** or **STAG2**. It is involved in sister chromatid cohesion and is responsible for 3D folding of the genome in interphase.

**Chromatin compartments** are 3D structures created by preferential self-interaction of active or inactive regions of the genome, creating A (active) and B (inactive) compartments.

**Condensin I and II** are protein complexes consisting of SMC2 and SMC4 and additional complex-dependent subunits. These complexes are primarily known for their role in chromosome condensation.

**CRISPR-GO** is a technique developed by Wang *et al*. (169) that uses a CRISPR/dCas9-based system to specifically and inducibly tether a specific region of the genome to certain structures within the nucleus, such as the nuclear lamina or Cajal bodies.

**CTCF** is a zinc-finger transcription factor that is able to stall the translocation of Cohesin along chromatin and functions to insulate regions of the genome.

**eRNAs** are noncoding RNAs transcribed from enhancers by RNA PolII.

**E-P loop** is a structure formed by bringing an enhancer in close spatial proximity to its promoter. Loops are proposed to be formed through the process of loop extrusion and mediated by the Cohesin complex and accessory proteins.

**LPS** is a molecule found on the outer membrane of bacteria and can be used to initiate an immune response in cells.

**Loop extrusion** is the proposed mechanism of E-P loop formation by the Cohesin complex. It involves threading a region of chromatin through Cohesin and a progressive increase in loop size as Cohesin translocates along chromatin.

**Mediator** is a protein complex containing up to 26 subunits and primarily functions as a channel of communication between transcription factors and RNA PolII.

**NIPBL/MAU2** heterodimer is involved in the loading of Cohesin onto chromatin.

**Nuclear lamina** is a network of filamentous lamin proteins and lamin-binding proteins that sits inside of the inner nuclear membrane. In addition to playing a structural role for the nucleus, it has multiple functional contributions, including to the control of transcription, DNA replication, apoptosis, cell cycle, and cell differentiation.

**Phase separation** is the concept behind the formation of membraneless organelles, which form from the aggregation of proteins and other macromolecules to give liquid-like structures known as “biomolecular condensates.”

**SPRITE** is a technique used to map genome-wide interactions (180). Unlike the 3C family of techniques, SPRITE does not depend on proximity ligation, and instead, DNA complexes that are crosslinked together are barcoded identically.

***Shh*** is a developmentally regulated gene that is involved in the patterning of a number of tissues during embryonic development. Tissue-specific enhancers control the expression of *Shh*, including the limb bud enhancer **ZRS** and the brain enhancer **SBE**. While not mentioned here, additional enhancers of Shh expression are the lung and gut enhancers MACS1 and SLGE (186), brain/floor plate enhancer SFPE (187), and the oropharyngeal epithelium enhancers MRCS1 and MFCS4 (188).

**TADs** are a feature of the 3D genome in which self-interaction is disproportionately favored. TAD boundaries are often defined by CTCF binding.

**TALEs** are DNA-binding proteins derived from *Xanthomonas* that can be customized to bind specific regions of DNA.

**VP64** and **VP128** are derivatives of the herpes simplex virus-1 transcription factor VP16. VP64 and VP128 contain 4X and 8X, respectively, tandem repeats of the minimal activation domain of VP16, creating a powerful transcriptional activator.

**WAPL** is responsible for the removal of Cohesin from chromatin.

## Conflict of interest

The authors declare that they have no conflicts of interest with the contents of this article.
